# Unveiling the oncogenic role of *PDK3* in head and neck squamous cell carcinoma, an integrative in silico and an in vitro approach

**DOI:** 10.1016/j.jgeb.2026.100768

**Published:** 2026-07-13

**Authors:** Aishath Shaheeda, Dharini N. Shetty, Padmanaban S. Suresh, Roshan Mascarenhas, Kabekkodu Shama Prasada

**Affiliations:** a Department of Cell and Molecular Biology, Manipal School of Life Sciences, Manipal Academy of Higher Education, Manipal, India; b School of Biotechnology, National Institute of Technology, Calicut 673601, Kerala, India; c Newcastle University Medicine Malaysia (NUMed), 79200 Johor Bahru, Malaysia.

**Keywords:** HNSCC, PDK3, Gene expression analysis, Pathway enrichment, Metabolic modeling, Survival prognosis

## Abstract

Head and neck squamous cell carcinoma (HNSCC) is a clinically aggressive malignancy with a poor prognosis. Emerging evidence highlights the importance of metabolic reprogramming in tumor progression and therapy resistance. However, the mechanisms driving metabolic adaptation in HNSCC remain incompletely understood. Here, we investigate pyruvate dehydrogenase kinase 3 (PDK3), a regulatory enzyme critical for aerobic glycolysis (the Warburg effect) in cancer cells. Using computational analysis and experimental validation, we explored PDK3’s role as an oncogene in HNSCC.

Our gene expression analysis revealed that PDK3 is significantly upregulated in tumors compared to normal tissues. Pathway enrichment analysis linked PDK3 to key metabolic pathways essential for cellular energy production and biosynthesis, including the TCA cycle, pyruvate metabolism, and glycolysis/gluconeogenesis. Additionally, PDK3 upregulation influenced immune cell distribution and drug response. Clinically, elevated PDK3 expression correlated with reduced overall survival in patients.

Functional validation in an HNSCC cell line (HSC3) demonstrated that PDK3 knockdown suppressed growth, proliferation, and migration, suggesting PDK3’s role as a potential tumor promoter in HNSCC. These findings indicate that targeting PDK3 could be a promising therapeutic strategy for HNSCC management.

## Graphical abstract

**Figure:**
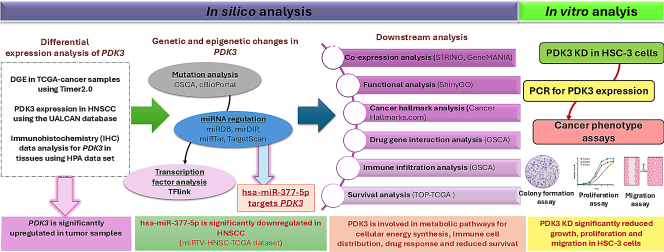

## 1 Introduction

Cancer remains a major global health challenge with approximately 9.7 million deaths worldwide, as reported by the International Agency for Research on Cancer (IARC) in 2022.^1^ Head and neck squamous cell carcinoma (HNSCC) arises from the mucosal epithelium of the upper aerodigestive tract, including the oral cavity, pharynx, and larynx.^2^ Despite significant advancements in diagnostic and therapeutic strategies, HNSCC continues to pose a substantial global burden, contributing markedly to cancer-related morbidity and mortality.^3^ According to GLOBOCAN 2020 estimates, HNSCC ranks as the seventh most common cancer worldwide, with approximately 890,000 new cases and 450,000 deaths annually. HNSCC accounts for 4.5% of total cancer incidence and 4.6% of cancer-related mortality globally.^4^ Anatomically, this estimate includes cancers of the lip and oral cavity (380,000 cases), larynx (185,000), nasopharynx (133,000), oropharynx (98,000), hypopharynx (84,000), and salivary glands (54,000).^4^ It is concerning that, the global burden of HNSCC has steadily increased from 1990 to 2021, with higher incidence observed among men, older individuals, and populations of lower socioeconomic status.^3^ A major clinical challenge in HNSCC is detection at advanced and more aggressive stages.^2^, ^3^ The major risk factors include tobacco, alcohol and HPV infections.^5^ Treatment strategies are largely stage-dependent and include surgery, radiotherapy, and systemic therapies such as chemotherapeutic agents and targeted immunotherapies.^2^ However, therapeutic resistance and disease recurrence remain significant obstacles to improved patient outcomes.^6^

In recent years, metabolic reprogramming has emerged as a hallmark of cancer, enabling tumor cells to rewire their metabolism in response to microenvironmental and therapeutic stress.^7^Metabolic reprogramming is characterized mainly by irregularities in the metabolism of glucose, glutamine, and lipids, among which alterations in glucose metabolism are most significant in tumor cells and are crucial in cancer progression and therapy resistance.^8^ The Warburg effect is a metabolic shift in cancer cells that promotes anaerobic glycolysis despite having efficient oxidative phosphorylation.^9^ The pyruvate dehydrogenase complex (PDC) has been reported to play critical role in energy metabolism by governings the conversion of pyruvate into acetyl-CoA for entry into the tricarboxylic acid (TCA) cycle. The activity of PDC is tightly regulated by the pyruvate dehydrogenase kinase (PDK) protein family, which phosphorylates the E1α subunit of PDC, thus inhibiting its function and promoting the Warburg effect.^10^

*PDK* exists in four isoforms: *PDK1, PDK2, PDK3*, and *PDK4.*^11^ Among the four isoforms, PDK3 has emerged as a particularly important regulator of proliferation, increased aggressiveness, and resistance to chemotherapy.^12^Higher levels of *PDK3* lead to a reduction in mitochondrial respiration activity, causing alterations in energy production. Investigations in different cancer cell lines have demonstrated that overexpression of *PDK3* results in increased glycolysis and resistance to drugs.^13^

Patients exhibiting high levels of *PDK3* have poor survival rates compared with those with lower *PDK3* levels.^14^ Furthermore, an increase in *PDK3* expression is correlated with negative pathological characteristics and poor cancer prognoses.^15^ Studies have also revealed significantly increased expression of *PDK3* in chemotherapy-resistant cancer cells, favoring the Warburg effect; conversely, suppressing *PDK3* has been shown to restore sensitivity to chemotherapy in gastric cancer.^16^ Aerobic glycolysis driven by *PDK1* and *PDK2* contributes to enhancing the stemness properties driven by TGFβ1 in head and neck cancer.^17^ This multifaceted tumor-promoting role of *PDK3* highlights its potential as a compelling target for therapeutic intervention in HNSCC.

Despite emerging evidence on the role of metabolic regulators in cancer progression, the specific role of *PDK3* in HNSCC remains to be elucidated. To bridge this knowledge gap, we conducted a comprehensive investigation via in silico and in vitro approaches to understand the oncogenic potential of PDK3 in HNSCC. Through systematic bioinformatic analyses, we evaluated *PDK3* expression patterns, regulatory mechanisms, prognostic significance, functional interaction networks, and associations with the tumor immune microenvironment and drug sensitivity. To validate these findings experimentally, we employed shRNA-mediated knockdown of *PDK3* in HNSCC cell lines (HSC3) and assessed its impact on key cancer phenotypes, such as cell growth, proliferation, and migration. This integrated analysis provides mechanistic insight into the role of PDK3 in HNSCC pathogenesis and supports its candidacy as a target for therapeutic intervention.

## 2 Methodology

### 2.1 Gene expression profiling and differential expression analysis

We investigated *PDK3* expression in tumor and normal tissue by using the TIMER 2.0 database (http://timer.cistrome.org/).^18^ We retrieved and visualized the transcriptomic data between tumor and normal tissues across 33 cancer types for *PDK3* expression, via the Gene DE module. The Gene DE module contains gene expression data collectively resourced from The Cancer Genome Atlas (TCGA) and Genotype-Tissue Expression databases (GTEx). The *PDK3* expression levels were represented as box plots in log2-transformed transcripts per million (TPM + 1) units. The statistical significance was evaluated using the Wilcoxon rank-sum test. We evaluated the protein level expression of PDK3 in normal and tumor tissues by immunohistochemistry (IHC) using the PDK3 specific antibody (Antibody HPA046583) available at the Human Protein Atlas (HPA) (https://www.proteinatlas.org/).^19^ The findings were further confirmed by using *PDK3* gene expression data in HNSCC, retrieved from the UALCAN portal (http://ualcan.path.uab.edu/).^20^ We accessed the TCGA RNA-sequencing data from the TCGA-HNSCC cohort and compared the data between normal tissues (*n* = 44) and primary tumor tissues (*n* = 520). The expression data was normalised as log2 TPM values, and statistical significance was determined by Student's *t-*test.A stagewise expression profile was also retrieved to determine whether the *PDK3* expression varies with tumor progression across clinical stages.

### 2.2 Analysis of genetic and epigenetic alterations of PDK3

Multiple publicly available databases were utilized to investigate the genetic and epigenetic alterations of PDK3 in HNSCC. Mutation frequency across different cancer types was initially assessed using the gene mutation module of TIMER 2.0 database (http://timer.cistrome.org/).^18^ The frequency and types of single-nucleotide variations (SNVs), including missense mutations, nonsense mutations, and frameshift deletions, as well as copy number variations (CNVs), were further analyzed using the Gene Set Cancer Analysis (GSCA) database (http://bioinfo.life.hust.edu.cn/GSCA/#/)^21^ based on the TCGA-HNSCC cohort. In addition, the association between CNVs and PDK3 mRNA expression was evaluated using Spearman's correlation analysis. Multiple testing correction was performed using the false discovery rate (FDR), and an adjusted *p*-value <0.05 was considered statistically significant.

### 2.3 miRNA-mediated posttranscriptional regulation of PDK3

To identify miRNAs that potentially target PDK3, a consensus approach was employed using multiple miRNA-target prediction databases, including the MicroRNA Target Prediction Database (miRDB) (https://mirdb.org/),^22^ microRNA Data Integration Portal (mirDIP) (https://ophid.utoronto.ca/mirDIP/),^23^ the miRTar database (https://miRTarBase.cuhk.edu.cn/)^24^ and TargetScan (https://www.targetscan.org/vert_80/).^25^ The predicted miRNAs from these databases were compared to identify the common miRNAs using the Venny 2.0 tool (https://bioinfogp.cnb.csic.es/tools/venny/index2.0.2.html). Only miRNAs that were commonly predicted across all four databases were selected for further analysis to ensure high-confidence target prediction. The differential expression of these candidate miRNAs in the TCGA-HNSCC dataset was evaluated using the miRTV tool (https://mirtv.ibms.sinica.edu.tw/index.php)^26^ and dbDEMC (https://www.biosino.org/dbDEMC/index)^27^ database. Transcription factors associated with the *PDK3* were identified using TFlink (https://tflink.net/protein/q15120/).^28^

### 2.4 Co-expression analysis and prediction of protein–protein interactions

To explore the potential interactome of *PDK3,* a comprehensive protein–protein interaction (PPI) network was constructed using the STRING (https://string-db.org/)^29^ and GeneMANIA (https://genemania.org/) databases.^30^ These databases integrate known and predicted protein interactions derived from the high-throughput experimental data. A default setting of medium confidence interaction score (>0.4) was applied in the STRING database to identify the significantly interacting proteins, whereas GeneMANIA integrates the multiple databases using an automatic weighting algorithm.^31^

### 2.5 Functional and cancer hallmark enrichment analysis

The biological role of *PDK3*-associated genes was investigated by performing functional enrichment analysis. The functional enrichment analysis consisted of Kyoto Encyclopedia of Genes and Genomes (KEGG) pathway prediction and Gene Ontology (GO) enrichment biological processes (BP), molecular functions (MF), and cellular components (CC)]. The GO enrichment testing was computed using ShinyGO (http://bioinformatics.sdstate.edu/go/) tool.^32^ Only the pathways exhibiting less than 0.05 false discovery rate were considered statistically significant.

In addition, the potential oncogenic processes connected with *PDK3* gene expression were explored through the Cancer Hallmarks platform (https://cancerhallmarks.com).^33^

### 2.6 Profiling of the tumor microenvironment and drug–gene interactions

Immunogenomic analysis was performed using the Gene Set Cancer Analysis (GSCA) (https://guolab.wchscu.cn/GSCA) database, which estimates the relative abundance of 24 immune cell types by integrating RNA sequencing and microarray data.^21^

In addition, potential drug that can target *PDK3* interactome network were queried using the GSCA Drug module (https://guolab.wchscu.cn/GSCA).^21^ This module incorporates pharmacogenomic datasets, including Genomics of Drug Sensitivity in Cancer (GDSC) and the Cancer Therapeutics Response Portal (CTRP).

### 2.7 Clinical correlation and survival analysis

We evaluated the prognostic relevance of PDK3 expression in HNSCC using the Tumor Online Prognostic Analysis Platform (http://biostatistics.online/),^34^ based on TCGA transcriptomic and clinical datasets. Patients were stratified according to the median expression level of *PDK3*. Overall survival was analyzed using the Kaplan-Meier method, and statistical significance was determined using the log-rank test.

### 2.8 Cell culture and transfection

HSC-3 cells were procured from the American Type Culture Collection. Cells were maintained in Dulbecco's Modified Eagle Medium (Himedia, India) supplemented with 10% fetal bovine serum (Capricorn, USA) and 1% penicillin-streptomycin (Himedia, India) at 37 °C in a humidified atmosphere containing 5% CO₂.

To generate the PDK3 knockdown cell line, HSC-3 cells (2 × 10^5^ cells/well) were seeded in 6-well plates and incubated for 24 h prior to transfection. Cells were transfected with PDK3-specific shRNA (sc-39,029-SH; Sigma, USA) using a lentiviral transfection reagent (MCE, USA) according to the manufacturer's instructions. Knockdown clones were selected using puromycin (1.0 μg/ml)^35^ for two weeks. A scrambled shRNA was used as a negative control.

Total RNA was isolated using RDP Trio™ reagent (HiMedia, India). Reverse transcription-quantitative PCR (RT-qPCR) was performed using 1000 ng RNA, and β-actin was used as an endogenous control. Relative gene expression was calculated using the 2^−^ΔΔCt method.^36^

### 2.9 Colony formation assay

Approximately 500 cells were seeded in 6 cm cell culture dishes and maintained in complete medium for 20 days. Colonies were fixed and stained with 0.5% crystal violet solution in methanol for 15 min. Excess stain was removed by PBS wash and the colonies were then air dried and counted for analysis.^36^

### 2.10 Proliferation assay

Approximately 5 × 10^4^ cells were seeded in 35 mm culture dishes. Cells were harvested at 24, 48, and 72 h, stained with trypan blue, and counted using a Neubauer hemocytometer following trypsinization. Cell proliferation was expressed as the total number of viable cells at each time point, and the number of cells in each interval is plotted as published earlier.^36^

### 2.11 Migration assay

Approximately 2.5 × 10^5^ cells were seeded in 6-well plate and cultured to 90% confluency. The cells were washed three times with PBS and incubated in serum-free medium for 24 h. A linear scratch was created at the centre of the cell monolayer using a sterile pipette tip. The cells were then washed to remove debris and maintained in complete medium until wound closure. Images were captured at specified time points using a CKX41 microscope (Olympus, Japan) connected to a camera (Jenoptik AG, Germany). ImageJ software (https://imagej.nih.gov/ij/download.html) was used to analyze the images.^37^

### 2.12 Statistical analysis

In vitro experiments were performed in three independent biological replicates. Quantitative data are presented as mean ± standard deviation (SD). Statistical comparisons between two groups were performed using a two-tailed Student's *t*-test, while multiple group comparisons were analyzed using one-way ANOVA. A *p*-value <0.001 (***) was considered statistically significant.

## 3 Results

### 3.1 High expression of *PDK3* in HNSCC

**Fig. 1:**
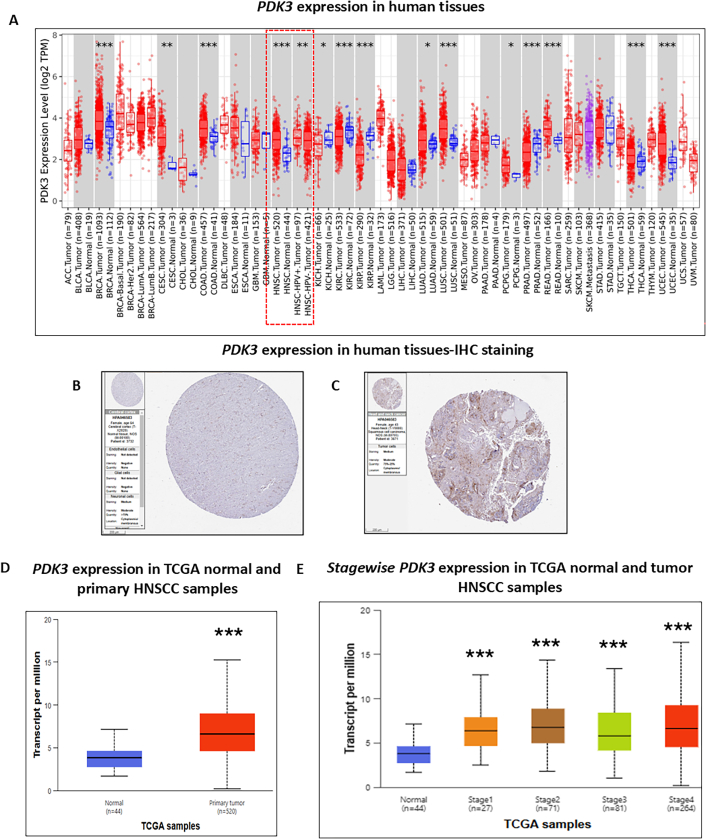
Gene expression profiling and differential expression analysis of PDK3: A. PDK3 gene expression across normal and cancer tissues, demonstrating elevated expression in multiple tumor types, visualized using the TIMER 2.0 database. B—C. IHC data retrieved from the HPA database showing higher expression of PDK3 in adenocarcinoma tissue (C) compared to normal brain tissue (B). D. PDK3 expression is significantly upregulated in primary tumor tissues compared to normal tissues based on TCGA data analysis using UALCAN database. E. Stagewise analysis showing differential PDK3 expression across tumor stages based on the TCGA dataset, using the UALCAN database. Data are represented as mean and SD, ***p ≤ 0.001 indicates statistical significance.

We explored *PDK3* expression levels across various cancer types compared with normal cells via the TIMER database (Fig. 1A), which revealed significant upregulation of the *PDK3* gene in HNSCC tumor samples, HNSCC-HPV-positive samples, and HNSCC-HPV-negative samples. Quantitative tissue (IHC) analysis using the HPA database (Fig. 1B–C) revealed that *PDK3* was highly expressed in HNSCC tissues than normal tissues, providing additional tissue-level confirmation of its expression. In addition, we explored the UALCAN database to visualize *PDK3* gene expression on the basis of the type of sample and individual cancer stage. The data revealed significant *PDK3* overexpression in the tumor samples (Fig. 1D). There was also an increase in *PDK3* expression as the cancer stage progressed from normal to stage 4 (Fig. 1E).

### 3.2 Genetic and epigenetic regulation of *PDK3* has heterogeneous results

**Supplementary Fig. 1:**
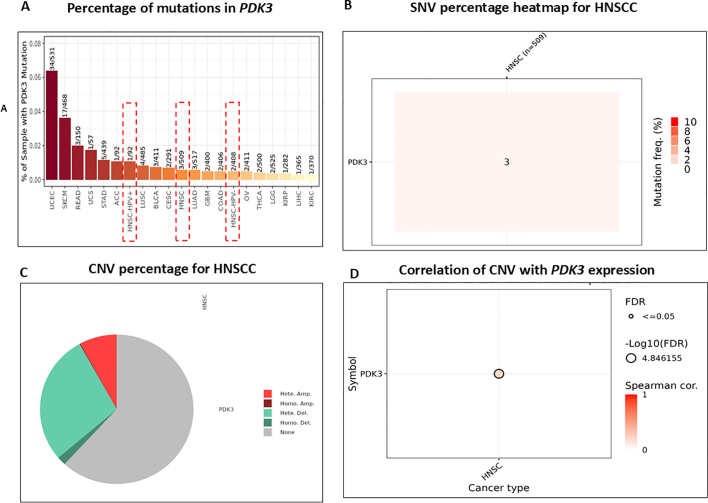
Genetic variation analysis of *PDK3:* A. *PDK3* mutation profile across cancers, visualized using the TIMER 2.0 database, represents a low overall mutation frequency with no dominant mutation class in HNSCC samples*.* B—C. The percentage and types of mutations observed at the *PDK3* locus in HNSCC confirm a minimal SNV burden and limited CNV events, with only a small proportion of heterozygous amplification or deletion. D. Correlation analysis between CNV status and *PDK3* expression in HNSCC, performed using the GSCA database, demonstrates no significant association, indicating that CNVs do not substantially contribute to *PDK3* dysregulation. E. Mutation landscape of PDK3 in HNSCC visualized using cBioPortal.

To understand the genetic basis of *PDK3* regulation, we evaluated multiple parameters, such as mutation frequency, somatic variation, and copy number variation, and their correlations with *PDK3* mRNA expression. The bar plot from the TIMER2.0 database for mutation frequency indicated a reduced level of *PDK3* mutation in TCGA cancer types at the *PDK3* locus (Supplementary Fig. 1A). The GSCA database revealed that approximately 3% of the somatic mutations in the TCGA patient cohort (Supplementary Fig. 1B) were not significantly related. Similar results were also obtained for CNV analysis (Supplementary Fig. 1C) (Supplementary Table 1). Correlation analysis between CNV and *PDK3* also revealed a nonsignificant relationship in HNSCC (Supplementary Fig. 1D). The mutation analysis revealed an insignificant relationship with *PDK3* OE under tumor conditions, which suggests the involvement of other regulatory events in gene expression.

**Fig. 2:**
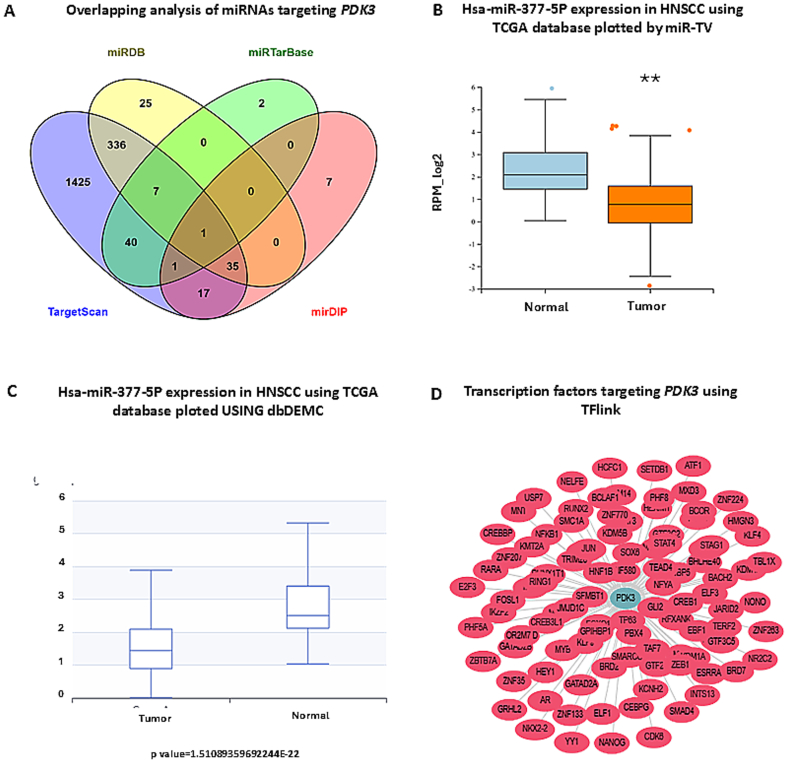
miRNA and transcription factor analysis of the *PDK3*: A. Overlapping analysis of miRNAs predicted to regulate *PDK3* across four publicly available databases. Hsa-miR-377-5p was predicted as a common target from all the four databases and its differential expression in HNSCC using B. miR-TV. C. dbDEMC databases demonstrated a significant downregulation in HNSCC TCGA tumor cohort compared to normal cohorts. D. Transcription factors regulating PDK3 expression were identified using TFlink. Data are represented as mean and SD, **p ≤ 0.01 indicates statistical significance.

We also performed miRNA screening to identify potential posttranscriptional regulators of *PDK3* via four established databases: TargetScan,^25^ miRTarBase,^24^ miRDB,^22^ and mirDIP.^23^ An overlap analysis conducted via the Venny 2.0 tool revealed hsa-miR-377-5p as a common miRNA predicted to target PDK3 across all four platforms (Fig. 2A). To assess its relevance in HNSCC, we examined the expression levels of hsa-miR-377-5p via the miRTV tool and further validated the findings via the dbDEMC database (Fig. 2B and C). Both datasets consistently revealed a significant downregulation of hsa-miR-377-5p in HNSCC tissues compared with normal controls, suggesting a potential loss of regulatory suppression of PDK3 in the tumor context. In addition, we used TFlink to visualize transcription factors associated with the *PDK3* gene, which identified 414 potential transcription factors associated with PDK3 posttranscriptional regulation (Fig. 2D).

### 3.3 Co-expression analysis of *PDK3* reveals relevant interaction partners

**Fig. 3:**
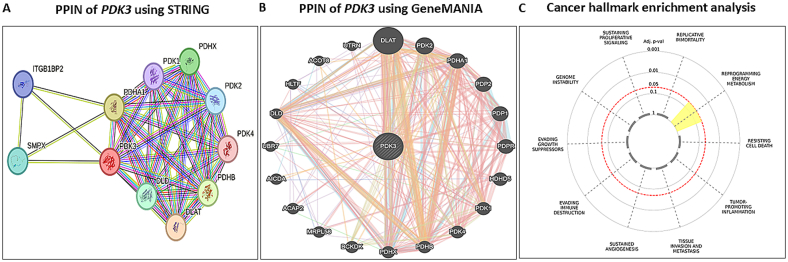
Coexpression and cancer hallmark enrichment analysis of the PDK3: PPIN analysis of *PDK3* using A. STRING and B. GeneMANIA databases demonstrated their potential functional interacting partners, most of which were crucial in metabolic reprogramming and tumorigenesis. C. Cancer hallmark enrichment analysis revealed that PDK3 is significantly associated with the reprogramming of energy metabolism.

Coexpression analysis of the *PDK3* gene was performed via STRING and GeneMANIA (Fig. 3A and B). STRING predicted the top 10 functional partners of *PDK3* genes, which include *DLAT, PDHA1, PDHB, PDHX, DLD, SMPX, PDK2, ITGB1BP2, PDK1, and PDK4,* which are involved in the metabolic reprogramming process (Fig. 3A). Genemania projected all the possible gene interactions with PDK3 (Fig. 3B). A cancer hallmark enrichment analysis of cancer hallmarks revealed that the *PDK3* gene was highly involved in the reprogramming of energy metabolism (Fig. 3C).

### 3.4 *PDK3* influences key signaling pathways in cancer progression

**Supplementary Fig. 2:**
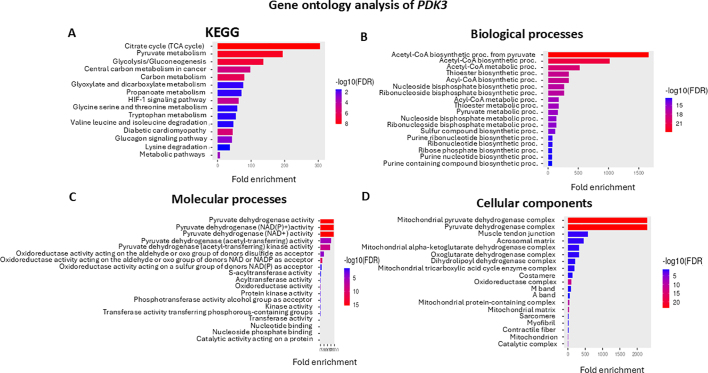
Functional enrichment analysis of *PDK3*-associated genes: Functional enrichment analysis of PDK3 associated genes demonstrates, A. significant perturbed pathways, identified by KEGG pathway enrichment analysis, highlighting enrichment of cancer-associated metabolic pathways. Gene ontology enrichment analysis showing B. Biological processes, C. Molecular functions and D. Cellular components reflecting altered metabolic activity and disrupted cellular energy homeostasis.

The ShinyGO tool was used to conduct GO analysis of *PDK3* to assess its biological attributes, which encompass the Kyoto Encyclopedia of Genes and Genomes (KEGG) pathway, biological processes (BP), molecular functions (MF), and cellular components (CC) (Supplementary Fig. 2A–D). The KEGG pathways identified included the TCA cycle, pyruvate metabolism, and glycolysis/gluconeogenesis (Supplementary Fig. 2A). The leading biological process (BP) terms identified were acetyl-CoA biosynthetic processes originating from pyruvate and acetyl-CoA biosynthetic processes (Supplementary Fig. 2B). Moreover, the most enriched molecular processes (MPs) included pyruvate dehydrogenase activity, pyruvate dehydrogenase activity [NAD(P)+], and pyruvate dehydrogenase activity (NAD+) (Supplementary Fig. 2C). The highly enriched cellular components were the mitochondrial pyruvate dehydrogenase complex and pyruvate dehydrogenase (Supplementary Fig. 2D).

### 3.5 *PDK3* expression influences immune cell abundance

**Fig. 4:**
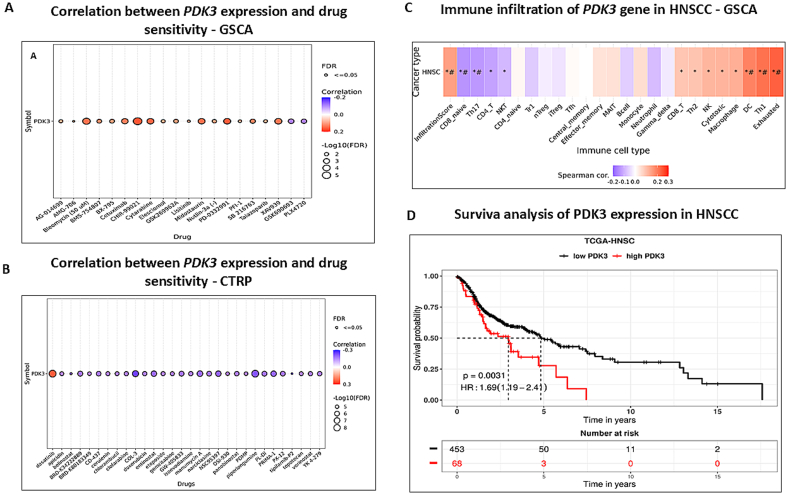
Tumor immune profiling, drug-gene interaction, and Survival analysis of *PDK3*: Correlation between *PDK3* expression and drug sensitivity based on A. GDSC and B. CTRP datasets retrieved from the GSCA database. C. Immune cell infiltration analysis showing the association between PDK3 expression and immune cell abundance in HNSCC, visualized using GSCA. D. Kaplan-Meier survival analysis demonstrating the association between *PDK3* and overall survival in HNSCC, demonstrating that higher PDK3 expression is associated with reduced overall survival in HNSCC patients, using the Tumor online prognostic analysis platform.

Immunogenomic analyses were performed via the GSCA tool. Spearman correlation analysis revealed the role of PDK3 expression in the TME (Fig. 4C). Higher *PDK3* expression in tumor cells significantly correlates with a reduction in naive and helper T cells and the accumulation of dysfunctional/exhausted effector cells. Although several antitumor immune subsets have been shown to be positively correlated, their cooccurrence with exhaustion markers implies a tolerogenic immune microenvironment, reflecting no reduction in the presence of immune cells but possibly impairing their effectiveness, thus promoting an immunosuppressive environment. This makes *PDK3* a candidate for therapeutic targeting, particularly in combination with immune checkpoint inhibitors, where reversing exhaustion is a key strategy.

### 3.6 Drug-gene interaction analysis

GDSC medications such as bleomycin, CHIR-99021, cytarabine, midostaurin, PD-0332991, and XAV939 are expected to be effective at killing cancer cells when the *PDK3* gene is active. However, GSK690693 and PLX4720 are predicted to be less effective at sensitising cancer cells expressing *PDK3* (Fig. 4A). Increased PDK3 expression was correlated with increased sensitivity to the CTRP drug dasatinib (Fig. 4B).

### 3.7 *PDK3* reflects a poor survival outcome

The Tumor Online Prognostic Analysis Platform was utilized to understand the associations between the expression of *PDK3* and survival outcomes. ToPP survival analysis revealed that increased expression of *PDK3* in HNSCC was associated with significantly poor survival outcomes (*p* = 0.0031) (Fig. 4D), indicating a potential prognostic impact of *PDK3* in HNSCC.

### 3.8 *PDK3* knockdown in HSC-3 cells

**Fig. 5:**
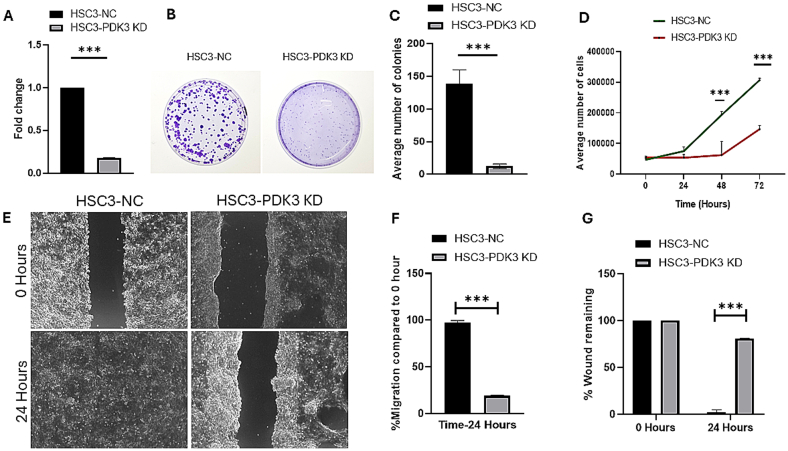
Functional validation of PDK3 knockdown in HSC3 cells: A. Relative PDK3 expression in HSC3 cells following shRNA-mediated knockdown compared to control cells. B. Representative images showing reduced colony formation ability in PDK3-knockdown (KD) cells compared to control vector (HSC3 NC)cells. C. Quantification of average number of colonies in PDK3-KD cells compared with control cells. D. Bar plot showing a reduced number of average proliferating cells in *PDK3*-KD HSC3 cells compared with control HSC3 NC cells at different intervals. E. Representative images of the wound healing assay showing a reduced migration ability of PDK3-KD HSC3 cells compared to control HSC3 NC cells. F. Quantification of the percentage of wound closure at 24 h compared with 0 h. G. Quantification of the percentage of the wound area remaining at 24 h. Data are presented as the mean and SD. *** p ≤ 0.001 indicates statistical significance.

To investigate the functional role of PDK3, shRNA-mediated knockdown of PDK3 was performed in HSC-3 cells. Knockdown efficiency was confirmed by RT-qPCR, which demonstrated a marked reduction in PDK3 expression compared with control (empty vector-transfected; HSC3-NC) cells (Fig. 5A).

We performed colony formation, proliferation, and wound healing assays to assess the impact of PDK3 silencing on key characteristics cancer cells such as growth, proliferation, and migration. The colony formation assay revealed a significant reduction in the number and size of colonies in PDK3 knockdown cells compared with HSC3-NC cells, indicating impaired clonogenic potential (Fig. 5B-C). Proliferation analysis demonstrated a significant decrease in cell proliferation in HSC3-PDK3 knockdown cells compared with control cells over 72 h (Fig. 5D). Furthermore, wound healing analysis showed a significantly reduced migratory capacity in PDK3 knockdown cells. While HSC3-NC cells exhibited rapid migration and nearly complete wound closure within 24 h. PDK3 knockdown cells displayed delayed migration, with a substantial wound area remaining unclosed (Fig. 5E-G). The data from colony formation, proliferation, and migration assay suggested that knock down of PDK3 significantly inhibited growth, proliferation, and migratory ability compared to vector transfected control cells. All experiments were performed in three independent biological replicates.

## 4 Discussion

Tumor metabolism is an actively explored field in tumorigenesis and therapy resistance.^38^ The ability of cancer cells to rewire their metabolic circuitry to enable better survival and fitness under stress makes it an important target for cancer studies.^39^ Otto Warburg in 1956 demonstrated that cancer cells rely on aerobic glycolysis even in the presence of sufficient oxygen to store their metabolic repository and energy sources for rapid growth and cell division.^9^ PDHC is a critical enzyme for decision-making in aerobic glycolysis and is masterly regulated by another group of enzymes called PDKs. The potential oncogenic properties of the 4 isoforms of PDKs have been investigated in multiple cancers.^11^, ^40^ Previous studies have indicated that *PDK3* is highly expressed in colon,^41^ lung,^42^ and urothelial cancers^15^and potentially plays a role in metabolic reprogramming and the advancement of tumors. However, the role of PDK3 as a potential oncogene in HNSCC has not been elucidated. Our comprehensive in silico and in vitro analyses demonstrated the importance of *PDK3* as a potential oncogene in HNSCC, and more investigations are needed to understand its clinical potential.

Our gene expression analyses revealed significant *PDK3* overexpression in HNSCC tumor samples. This observation is consistent across several datasets, including TIMER2.0 and the Human Protein Atlas, thereby strengthening the evidence for the potential role of PDK3 as an oncogene in HNSCC. Research has indicated that there is a strong link between human papillomavirus and the development of head and neck cancer.^43^ Our findings from the TIMER database indicate that both HPV-positive and HPV-negative HNSCC tumors present increased *PDK3* expression, suggesting that this upregulation is not exclusively reliant on HPV infection and points to a more extensive role for *PDK3* in HNSCC development. The increased *PDK3* expression in tumor samples from the UALCAN database underscores its involvement in oncogenesis. Consistent with this finding, an increase in PDK3 expression was also found with increasing cancer stage. This finding aligns with earlier research showing that the expression level of *PDK3* increased in colon cancer patients with increasing cancer stage.^15^, ^44^

Our investigation of genetic alterations revealed a low mutation frequency of *PDK3* in HNSCC, suggesting that mutations at the *PDK3* locus are not common drivers of this type of cancer. The mutation frequency of PDK3 was 0.589% in HNSCC, 1.08% in HPV-positive HNSCC, and 0.490% in HPV-negative tumors. However, the somewhat higher mutation frequency among HPV-negative patients than among HPV-positive patients warrants further exploration. This finding, considering the significant alterations in *PDK3* expression, indicates that the upregulation of *PDK3* in HNSCC is likely attributed to regulatory changes rather than genetic mutations within the *PDK3* gene itself. Copy number variations (CNVs) were heterozygous with heterozygous amplifications and deletions. Most of the samples did not present mutations.

We also examined the miRNAs that target *PDK3* via multiple databases, such as the miRDB,^22^ mirDIP,^23^ TargetScan,^25^ and miRTar^24^ databases. We identified hsa-miR-377-5p as a common target between these databases. We subsequently assessed the expression of this miRNA and found that its expression is significantly downregulated in HNSCC. Previous studies in other cancers, such as lung^45^ and liver^46^ cancer, revealed significant downregulation of hsa-miR-377-5p, and its overexpression reduced cancer properties. These findings indicate that *PDK3* expression is strictly regulated by posttranscriptional regulation in HNSCC. We conducted a transcription factor analysis via TFlink and identified the transcription factors associated with the *PDK3* gene. The *PDK3* gene was found to be associated with 414 transcription factors.

Our network analysis performed with STRING and GeneMANIA identified critical proteins that interact with *PDK3*, providing insights into its functional relationships. The network analysis revealed several functional partners of *PDK3*, including *DLAT*, *PDK2*, *PDHA1*, *PDK4, PDHB*, *PDHX*, *DLD*, *SMPX*, *PDK1* and *ITGB1BP2*. and. These partners form a complex network involved in energy metabolism and other cellular activities. The co-expression of *PDK3* with multiple components and regulators of the pyruvate dehydrogenase complex highlights a coordinated approach by tumor cells to effectively manage pyruvate metabolism and energy production. Cancer hallmark enrichment analysis suggested that the reprogramming of energy metabolism, a significant hallmark linked to *PDK3*, plays a critical role in the specific metabolic adaptations noted in cancer cells.^47^, ^48^, ^49^

According to ShinyGO functional enrichment analysis, *PDK3* is involved in several critical metabolic processes, such as glycolysis/gluconeogenesis, pyruvate metabolism, and the TCA cycle. Dysregulation of these pathways is a hallmark of cancer and is essential for the biosynthesis and generation of cellular energy.^48^ The presence of *PDK3* in these pathways supports its involvement in the metabolic reprogramming typical of cancer cells. Its role in acetyl-CoA biosynthetic processes and its connection to the mitochondrial pyruvate dehydrogenase complex further highlight its importance in cellular metabolism and energy homeostasis. These findings are consistent with the established function of *PDK3* in regulating pyruvate dehydrogenase activity, a vital enzyme in glucose metabolism.^11^

Our immunogenomic analyses revealed that *PDK3* expression is related to alterations in the tumor immune microenvironment. *PDK3* expression was positively correlated with the overall immune infiltration score as well as with pro-tumorigenic and immune-suppressive cell types, including exhausted T cells, dendritic cells (DCs), Th1 cells, and macrophages. Research has demonstrated that macrophage profiling in head and neck cancer can significantly improve patient prognosis, highlighting the critical role of TAMs in disease progression and therapeutic response.^50^ Research has demonstrated that higher levels of cytotoxic CD8^+^ T lymphocytes are associated with improved prognosis in oropharyngeal cancer.^51^ Conversely, PDK3 expression was negatively correlated with naive CD8+ T cells, NK cells, and Th17 cells, which are typically associated with antitumor immunity. This inverse association suggests that elevated PDK3 expression may contribute to immune evasion mechanisms in HNSCC. Furthermore, evidence indicates that PDK3-mediated metabolic reprogramming may limit nutrient availability to tumor-suppressive immune cells, thereby reducing their cytotoxic efficiency against cancer cells.^52^ Interestingly, several antitumor immune cells are positively correlated; however, the presence of exhaustion markers indicates a reduction in their efficiency in the tumor microenvironment and promotes an immunosuppressive environment.

Drug–gene interaction analysis highlighted several potential drug targets related to *PDK3*. Drugs such as bleomycin, CHIR-99021, cytarabine, midostaurin, PD-0332991, and XAV939 are likely to be effective against cancer cells with elevated *PDK3* expression suggests that targeting *PDK3* could be therapeutically beneficial. Additionally, the link between high expression of *PDK3* and increased sensitivity to the CTRP drug dasatinib further underscores the viability of *PDK3* as a target in cancer therapy. Dasatinib has also been shown to be effective in the treatment of multiple cancers.^53^

Our survival analysis from Tumor Online Prognostic Analysis Platform revealed that high *PDK3* expression is linked to poor survival outcomes in cancer patients. These findings suggest that *PDK3* might be a potential prognostic biomarker, indicating that patients with tumors that have high *PDK3* expression may experience less favorable clinical outcomes. Research indicates that individuals with higher levels of *PDK3* exhibit lower overall survival rates than those with lower *PDK3* levels.^54^ Furthermore, increased *PDK3* expression is linked to adverse pathological characteristics and poor cancer prognosis, highlighting its potential as a marker for urothelial cancer.^15^ Elevated levels of *PDK3* are strongly associated with DNA repair and replication.^15^

The experimental validation of our in silico studies was performed via targeted knockdown of the PDK3 gene in HSC3 oral squamous carcinoma cells, and the functional role of PDK3 was evaluated via phenotypic assays. Consistent with our in silico analysis, we observed a marked reduction in cell proliferation, growth rate, and migration upon PDK3 knockdown. These findings support the hypothesis that PDK3 promotes tumorigenic behavior in HNSCC through its regulatory effects on mitochondrial metabolism and glycolytic flux. By phosphorylating and inactivating PDHC, PDK3 diverts pyruvate away from oxidative phosphorylation to metabolically stimulate aerobic glycolysis, thereby sustaining the biosynthetic demands of rapidly dividing tumor cells.^10^ PDK3-knockdown HSC3 cells exhibited a marked reduction in migratory capacity, highlighting the role of PDK3 in epithelial-mesenchymal transition (EMT), cytoskeletal reorganization, and extracellular matrix remodeling. This finding is consistent with previous reports indicating that PDK3 expression is negatively correlated with epithelial markers and positively correlated with mesenchymal markers in oral squamous cell carcinoma. Furthermore, PDK3 knockdown significantly reduced cell growth and invasion, whereas its overexpression enhanced these properties.^54^

Overall, these in vitro observations corroborate our in silico findings, highlighting the importance of PDK3 as a central driver of metabolic reprogramming and malignant progression in HNSCC.^17^ Importantly, these results also underscore the translational importance of PDK3 as a candidate biomarker and therapeutic target. Targeting PDK3-mediated metabolic pathways may enhance the efficacy of existing chemotherapeutic agents, particularly in tumors exhibiting a glycolytic phenotype.^55^ However, therapeutic strategies targeting PDK3 must be approached with caution, as PDK3 play critical role in maintaining energy homeostasis in high-energy demand tissues such as heart and skeletal muscles, and its inhibition may cause systemic toxicity.^56^

Future studies should explore the broader impact of PDK3 on tumor–immune interactions and treatment resistance, particularly in the context of the tumor microenvironment. Additionally, as PDK3 belongs to a family of four highly homologous isoforms (70%), potential functional redundancy and compensatory mechanisms among PDK isoforms influence therapeutic efficacy and durability, warranting further investigation. Taken together, our findings provide a compelling rationale for the continued investigation of PDK3 in the pathogenesis and treatment of HNSCC.

## 5 Conclusion

Our study used computational analysis and experimental validation to investigate the role of *PDK3* in HNSCC. Data on *PDK3* gene expression revealed that it was overexpressed, suggesting that it might play an oncogenic role. We explored the genetic, epigenetic, and posttranscriptional regulation of *PDK3*. Downstream pathways, clinical outcomes, and microenvironment reprogramming are also perturbed upon PDK3 OE in HESCs. Potential therapeutic compounds that could be used to treat HNSCC were identified by our investigation. The results of the experimental validation and computational analysis indicate the potential oncogenic role of PDK3 in HNSCC. These findings warrant further exploration of the therapeutic effects of PDK3.

The following are the supplementary data related to this article.

Supplementary Table 1 The percentages and types of SNVs and CNVs at the *PDK3* locus in HNSCC from the GSCA database.

## CRediT authorship contribution statement

**Aishath Shaheeda:** Writing – review & editing, Writing – original draft, Visualization, Validation, Methodology, Investigation, Formal analysis, Data curation. **Dharini N. Shetty:** Writing – original draft, Visualization, Validation, Software, Methodology, Investigation, Formal analysis, Data curation. **Padmanaban S. Suresh:** Writing – review & editing. **Roshan Mascarenhas:** Writing – review & editing. **Shama Prasada Kabekkodu:** Writing – review & editing, Visualization, Validation, Supervision, Software, Resources, Project administration, Methodology, Investigation, Funding acquisition, Formal analysis, Data curation, Conceptualization.

## Ethics statement

The authors declare that they have no ethical statements to disclose since the research does not include any animal or human studies.

## Declaration of generative AI and AI-assisted technologies in the writing process

During the preparation of this work, the authors used the Rubriq AI tool for language editing. After using this tool, the authors reviewed and edited the content as needed and take full responsibility for the content of the publication.

## Declaration of competing interest

The authors declare that they have no known competing financial interests or personal relationships that could have appeared to influence the work reported in this paper.

## Data Availability

The authors declare that all data used in this study were obtained from publicly available datasets.
