# Supplementary material for: Unveiling the oncogenic role of PDK3 in head and neck squamous cell carcinoma, an integrative in silico and an in vitro approach
Source: J Genet Eng Biotechnol. 2026 Jul 13;24(3):100768. doi: 10.1016/j.jgeb.2026.100768 (PMC13382302; doi:10.1016/j.jgeb.2026.100768)
Supplement: Supplementary Table 1 — The percentages and types of SNVs and CNVs at the PDK3 locus in HNSCC from the GSCA database. [file mmc3.docx]

| **Percentages and types of SNVs and CNVs at the PDK3 locus HNSCC** | | | | | | |
| --- | --- | --- | --- | --- | --- | --- |
| **SNVs** | | | | | | |
| **Cancer type** | **Effective mutations** | | **Non effective mutations** | **Sample size** | | **Percentage** |
| **BRCA** | 3 | | 2 | 509 | | 0.589390963 |
| **CNVs** | | | | | | |
| **Cancer type** | **Total amplification**  **(%)** | **Total**  **Deletion**  **(%)** | **Heterozygous amplification**  **(%)** | **Heterozygous deletion**  **(%)** | **Homozygous amplification (%)** | **Homozygous deletion**  **(%)** |
| HNSCC | 8.045977 | 30.0766284 | 7.8544061 | 28.1609195 | 0.1915709 | 1.9157088 |
